# Rett Syndrome Spectrum in Monogenic Developmental-Epileptic Encephalopathies and Epilepsies: A Review

**DOI:** 10.3390/genes12081157

**Published:** 2021-07-28

**Authors:** Carlotta Spagnoli, Carlo Fusco, Francesco Pisani

**Affiliations:** 1 Child Neurology Unit, AUSL-IRCCS di Reggio Emilia, 42123 Reggio Emilia, Italy; carlo.fusco@ausl.re.it; 2 Child Neuropsychiatry Unit, University-Hospital of Parma, 43123 Parma, Italy; francesco.pisani@unipr.it

**Keywords:** epilepsy, epileptic and developmental encephalopathies, Rett syndrome, Rett syndrome spectrum, Rett-like, stereotypies, movements disorders, neurogenetics

## Abstract

Introduction: Progress in the clinical application of next-generation-sequencing-based techniques has resulted in a dramatic increase in the recognized genetic heterogeneity of the Rett syndrome spectrum (RSS). Our awareness of the considerable overlap with pediatric-onset epilepsies and epileptic/developmental encephalopathies (EE/DE) genes is also growing, and the presence of variable clinical features inside a general frame of commonalities has drawn renewed attention into deep phenotyping. Methods: We decided to review the medical literature on atypical Rett syndrome and “Rett-like” phenotypes, with special emphasis on described cases with pediatric-onset epilepsies and/or EE-DE, evaluating Neul’s criteria for Rett syndrome and associated movement disorders and notable stereotypies. Results: “Rett-like” features were described in syndromic and non-syndromic monogenic epilepsy- and DE/EE-related genes, in “intellectual disability plus epilepsy”-related genes and in neurodegenerative disorders. Additionally, prominent stereotypies can be observed in monogenic complex neurodevelopmental disorders featuring epilepsy with or without autistic features outside of the RSS. Conclusions: Patients share a complex neurodevelopmental and neurological phenotype (developmental delay, movement disorder) with impaired gait, abnormal tone and hand stereotypies. However, the presence and characteristics of regression and loss of language and functional hand use can differ. Finally, the frequency of additional supportive criteria and their distribution also vary widely.

## 1. Introduction

Rett syndrome (RTT) was first described in the 60s, and then atypical forms were identified. Updated diagnostic criteria were developed in 2010 in an effort to avoid diagnostic confusion (Table 1) [1]. The term “Rett-like” spread in the medical literature to describe patients presenting with overlapping features but not satisfying criteria either for typical or atypical RTT [2]. The dramatic evolution in the number of potentially involved genes, paralleling the advent of next-generation-sequencing (NGS)-based techniques, promoted the term “Rett syndrome spectrum (RSS)”, in order to better account for heterogeneity [2]. Following this input from implemented genetic testing, there has been renewed interest in distinguishing clinical features, in order to enhance diagnostic accuracy and reduce pitfalls in data interpretation.

As the rate of developmental and epileptic encephalopathies (DE/EE) genes causing complex neurological phenotypes with reported “RTT-like” features is high, we reviewed the literature with the aim of describing the clinical and genetic characteristics of patients harboring pathogenic or likely pathogenic variants in genes causative of DE/EE and epileptic phenotypes and described as having a “RTT-like” or atypical Rett phenotype, in order to describe how this label is used in clinical and research practice with respect to current diagnostic criteria (Neul’s criteria) and what underlying conditions have been most frequently implicated. In order to highlight their similarities and differences from typical and atypical Rett, these conditions will also be summarized in the first part of this review.

## 2. Typical RTT

RTT is an X-linked disorder characterized by four clinical stages. Following normal early development, head growth often decelerates (between 2–4 months), followed by psychomotor and purposeful hand movements regression (6–12 months). Between 1 and 3 years, autistic features, intellectual disability (ID), hand stereotypies (HS), abnormal gait, motor dysfunction and breathing abnormalities take shape, followed by stabilization or recovery [3,4,5,6]. If no sign of regression develops by 5 years, diagnosis should be questioned [1]. Later motor deterioration (teenage years) brings spasticity, dystonia, scoliosis and/or parkinsonism (Table 2).

### 2.1. Epilepsy

Epilepsy is present in 60–80% of patients, usually from the second-third stage [3]), with no typical semiology at presentation. Onset before 1 year is atypical [4], while onset after 5 years predicts seizure control [5]. Focal-onset and bilateral tonic-clonic seizures are frequent, as are early-life febrile seizures [3,5]. Generalized seizures correlate with drug-resistance [3] EEG parallels clinical changes (Table 2) [5,6].

### 2.2. Movement Disorder (MD) and Stereotypies

In younger patients, hyperkinetic MD is prominent, with ataxia, dystonia, chorea, myoclonus and, most typically, hand stereotypies (HS). These coincide with, or precede the loss of purposeful hand movements in 60% of patients harboring *MECP2* variants. Individuals with typical RTT have the highest frequency and earliest onset. Hand mouthing and clapping/tapping are frequent (more than wringing/washing). While hand function decreases over time, HS prevalence and frequency remains relatively unchanged, although variety decreases. Stereotypies other than manual tend to disappear with age [9].

### 2.3. Genotype-Phenotype Correlations

Pathogenic variants in the methyl-CpG binding protein 2 (*MECP2*) gene account for 95% of typical RTT [10]. Occasionally associated genes include Huntingtin (*HTT*), SWI/SNF Related, Matrix Associated, Actin Dependent Regulator Of Chromatin, Subfamily A, Member 1 (*SMARCA1*), Zinc finger protein 238 (*ZNF238*) [11], Succinate Dehydrogenase Complex Flavoprotein Subunit A (*SDHA*), Kinesin Family Member 1A (*KIF1A*) [12], γ-Aminobutyric Acid Type A Receptor Subunit Beta2 (*GABRB2*) [13], potassium voltage-gated channel subfamily B member 1 (*KCNB1*) [2] and Jumonji Domain Containing 1C (*JMJD1C*) [14].

## 3. RTT Variants

### 3.1. From “Early-Onset Seizure” Variant to “CDKL5-Related” Disorder

Epilepsy before 1 year of age is present in almost all patients carrying cyclin-dependent kinase-like 5 (*CDKL5*) variants who are affected by epilepsy [3,4]. Early epilepsy with normal interictal EEG and severe hypotonia are key early clinical features [15]. Neurological examination reveals poor eye contact as early as epilepsy onset [16]. Some RTT features (head growth deceleration, stereotypies, hand apraxia) become more evident in older, ambulatory patients. Other RTT signs (nearly normal early development followed by regression, with the loss of fine finger skills and intense eye communication) are absent [15].

### 3.2. Epilepsy

Typical clinical and EEG semiology can be identified, namely the peculiar tonic-vibratory focal seizures followed by a series of spasms and an initially normal EEG that later deteriorates (Table 2) [4,16,17,18].

### 3.3. From Congenital RTT Variant to FOXG1 Syndrome

Intragenic variants or duplications/deletions of the Forkhead Box G1 (*FOXG1*) gene have been reported in the neurodevelopmental disorder (NDD) initially classified as “congenital RTT variant”. Afterwards, differing clinical features were recognized as sufficiently distinct to delineate “*FOXG1* syndrome” [8], which is more severe than RTT with respect to ambulation, reciprocity, receptive language and sleep disorder, and lacking regression. In total, 9.5% of *FOXG1*-positive females and less than 5% of males fulfill the diagnostic criteria for RTT [19].

Two distinct phenotypes exist based on the presence of deletion/intragenic variant versus duplication. In the first case, there is a complex NDD featuring acquired microcephaly, epilepsy, motor and cognitive delay, severe ID, a dyskinetic disorder and structural brain abnormalities (corpus callosum hypoplasia, cortical thickening or a simplified gyral pattern) [7,8,20]. Neurodevelopmental delay is a presenting feature, often accompanied by poor feeding, irritability, hypotonia, and visual inattention [7]. Severe postnatal microcephaly (−4 to −6 SD) becomes apparent after 1 month [19]. DD is severe-to-profound: patients are usually non-ambulant with severely impaired functional hand use [21]. Rare descriptions of a milder phenotype, with independent ambulation, spoken language, and normocephaly are reported with missense variants. Hyperkinetic movements are a major clinical feature [7].

Differently, duplications of 14q12 often present with infantile spasms, have ID with major speech involvement [22] and autistic features. Almost all have normal head circumference before 3 years [17].

Other genes causing the “early seizure variant” include syntaxin-binding protein 1 (*STXBP1*) [23] and Sodium channel, voltage gated, type VIII, α subunit (*SCN8A*) [24,25].

### 3.4. Epilepsy

Epilepsy is diagnosed in 78–87% of subjects with *FOXG1*-related disorders [19,21]. 

Patients with deletions/intragenic variants usually have their epilepsy onset within the second year [19], with a variety of epilepsy types (Table 2). Drug resistance is common [4,21].

In contrast, subjects with *FOXG1* duplications present with infantile spasms (mean age at onset: 7.4 months) [21]. Most also exhibit focal seizures (onset: 5 months–6 years), often in association with spasms [20]. In a minority of cases, tonic or myoclonic seizures recur later on [21]. Electro-clinical characteristics are detailed in Table 2.

### 3.5. MD and Stereotypies

A wide variety of MDs has been identified, most commonly dystonia (76%), choreoathetosis (88%) and orolingual/facial dyskinesias (80%). Ninety-three percent of patients have a mixed MD [7]. Continuous dyskinesia with a mixture of hyperkinetic, dystonic, choreic and athetoid movements, mainly involving limbs and face, and stereotypic movements impairing purposeful hand use and fine motor skills are typical [20].

Stereotypies are present in 50% of patients: mouthing of toys, grasping, nail biting and, rarely, midline wringing [19]. Stereotypies in the lower limbs (pulling, pedaling), trunk (body rocking) and bruxism are less frequent [7].

Patients carrying the 14q12 duplication have mild hyperkinetic and perseverative, stereotyped hand–mouth movements [20].

Abnormal movements’ onset falls within the first year of age [7], usually between the fifth and twelfth month. No change in severity or semiology is observed over time [20]. Additional genes reported with atypical/variant RTT include γ-Aminobutyric Acid Type B Receptor Subunit 2 (*GABBR2*), Potassium voltage-gated channel subfamily A member 2 (*KCNA2*), Histone Deacetylase 4 (*HDAC4*), Netrin G1 (*NTNG1*) and three genes not yet associated with human disease when reported: annexin A11 (*ANXA11*), kinesin family member 4B (*KIF4B*) and RNA-polymerase I-specific transcription initiation factor 3 (*RRN3*) [26]. *ANXA11* is now considered an amyotrophic lateral sclerosis (ALS types 1 and 23) gene, and no further reports linked it to RSS. We found no further clinical reports pertaining to *RRN3* or *KIF4B*.

## 4. RETT-Like Phenotypes

Apart from a minority of patients harboring *MECP2* [27] and *FOXG1* [28] variants, which will not be covered further in this review, overlapping RTT-like features have been described in many DE/EE or epilepsy genes. These can be clinically divided into syndromic versus non-syndromic monogenic [2]. Due to their clinical relevance in the differential diagnosis, neurodegenerative conditions will also be reviewed. 

### 4.1. Syndromic Conditions

#### 4.1.1. Pitt–Hopkins Syndrome (TCF4) and Pitt-Hopkins-Like Syndrome (CNTNAP2, NRXN1)

Pitt–Hopkins syndrome (PHS) is a NDD with distinctive facial dysmorphisms and overbreathing, severely impaired speech and (in some patients) progressive microcephaly and epilepsy. Ataxic gait and stereotypies (hand clapping) are frequent. The classical phenotype is mainly caused by heterozygous pathogenic transcription factor 4 (*TCF4*) variants. Sporadic “RTT-like” patients with hand wringing [2] and atypical RTT with unspecified HS have been reported [11,29].

No cases with atypical RTT/RTT-like phenotypes have been described with bi-allelic loss of function variants in Contactin-associated protein-like 2 (*CNTNAP2*) and neurexin-1-α (*NRXN1*) genes.

#### 4.1.2. Cornelia de Lange (CdL) Syndrome (SMC1A, HDAC8, NIPBL, SMC3, RAD21)

This syndrome is characterized by ID, dysmorphic facial features, hirsutism, short neck, major malformations (especially limb defects) and can be caused by pathogenic variants in five genes. X-linked pathogenic variants in the structural maintenance of chromosomes 1A (*SMC1A*) are associated with epilepsy in 45% of cases, usually generalized, with onset in the first year of life in nearly 90% of cases [30]. Importantly, limb defects are rare. Clinical phenotype can include stereotypic movements [30,31,32] and hand stereotypies, microcephaly, in some cases regression, and rarely abnormal breathing, resulting in a clinical diagnosis of typical RTT [2] or “RTT-like” in single patients [30]. A single patient harboring a novel, de novo pathogenic variant in the X-linked histone deacetylase 8 (*HDAC8*) gene showed early-onset DD, loss of purposeful hand movements, psychomotor deterioration (aged 6 years), distinctive facial features and multiple congenital anomalies reminiscent of CdL. She also had epilepsy. Four main revised criteria, two exclusion criteria and 10/11 supportive criteria classify her as RTT-like [33]. No cases with a clinical diagnosis of “Rett-like” features have been reported in association with pathogenic variants in nipped-B-like (*NIPBL*), structural maintenance of chromosomes 3 (*SMC3*) or cohesin complex component (*RAD21*).

#### 4.1.3. Phelan–McDermid Syndrome (SHANK3)

Autosomal dominant ID with speech impairment, hypotonia, epilepsy, autism, dysmorphic features and renal and/or structural brain abnormalities have been described in association with either intragenic variants or deletions involving the SH3 and multiple ankyrin repeat domains 3 (*SHANK3*) gene. However, one patient classified as RTT [34] and two as RTT-like [2] were reported.

#### 4.1.4. Christianson Type X-Linked Mental Retardation Syndrome (SLC9A6, NHE6)

It is an X-linked syndromic form of profound ID with absent speech, epilepsy in 80% of cases, frequent hyperkinetic MD (ataxic gait) with cerebellar atrophy, acquired microcephaly, happy demeanor and autistic features. Regression has been seldom reported. Two cases with RTT-like features and pathogenic variants in the Sodium-hydrogen exchanger 6 (*NHE6*) gene have been described [35,36], while no cases have been so far reported with solute carrier family 9 member 6 (*SLC9A6*) gene variants.

#### 4.1.5. Glass Syndrome (SATB2)

Although heterozygous disease-causing variants in the Special AT-rich sequence-binding protein 2 (*SATB2*) gene are associated with Glass syndrome (NDD, speech impairment, non-specific dysmorphic features and, frequently, cleft palate or teeth abnormalities, with infrequent epilepsy and no regression), two RTT-like cases have been described, without seizures or regression. The neurobehavioral profile was characterized by the absence of purposeful hand movements, HS (wringing and tapping), poor sleep, ID and awake bruxism. Brain MRI showed a simplified fronto-temporal gyral pattern in one, while EEG revealed increased high-amplitude delta activities in one patient and focal spike-and-wave complexes in the second [37].

#### 4.1.6. HNRNPU-Related Disorders

Heterogenous nuclear ribonucleoprotein U (*HNRNPU*) heterozygous pathogenic variants cause a DE/EE that can present with associated dysmorphic features and cardiac and renal involvement [38], although patients without overt dysmorphisms have also been described. Autistic features, prominent stereotypies [38,39,40] and, occasionally, abnormal breathing patterns reminiscent of RTT and PHS have been reported in single patients [39,40].

#### 4.1.7. MEIS2

Meis Homeobox 2 (*MEIS2*)-related disorder is an autosomal dominant syndromic form of ID, characterized by ASD, minor dysmorphisms, atrial sept defects, ventricular septal defects, oro-facial clefting and bifid uvula. One patient with epilepsy, HS and 3/11 Neul’s supportive criteria (impaired sleep, abnormal tone, intense eye communication) had a RTT-like diagnosis [41].

## 5. Monogenic Non-Syndromic Conditions

### 5.1. DE/EE Genes

**Figure 1:**
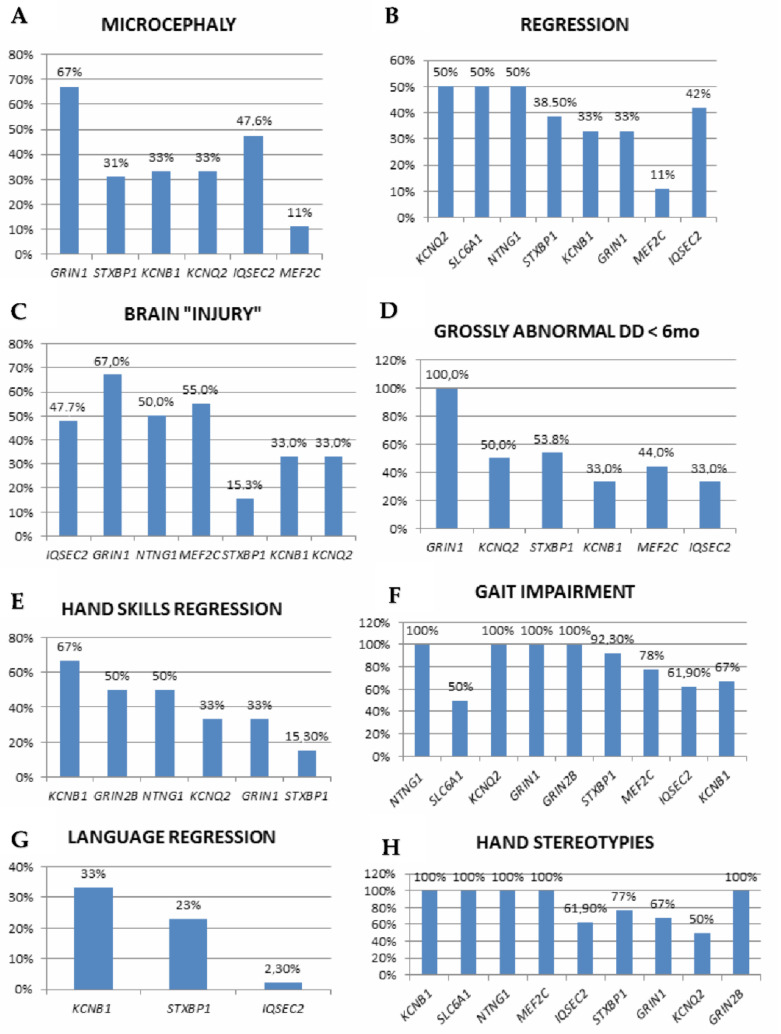
Neul’s inclusion and exclusion criteria applied to the reviewed cases according to genetic diagnosis (only genes with more than one reported case are shown), (**A**–**H**): microcephaly, regression, brain “injury”, grossly abnormal development in the first 6 months, hand skills regression, gait impairment, language regression, hand stereotypies.

**Figure 2:**
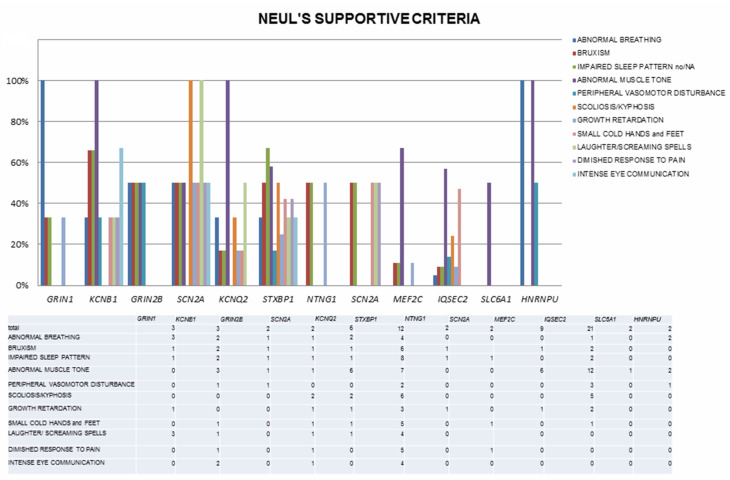
Neul’s supportive criteria in the cohort of reviewed cases, depicted according to genetic diagnosis (only genes with more than one reported case are shown).

The involved genes are mainly linked to three major pathways: synaptic function (including ion homeostasis, synaptic vesicle trafficking, and synaptic function for GABAergic, glutamatergic or dopaminergic transmission), chromatin modulation and ubiquitin conjugation [42]. Associated OMIM diseases, clinical diagnoses within the RSS and a number of included cases are listed in Table 3, while detailed clinical, EEG and genetic findings are reported in Table S1. Neul’s criteria are depicted in Figure 1 and Figure 2 and in Table S1.

### 5.2. STXBP1

Single RTT-like patients have been reported [11,27,29,35]. They mostly fulfill criteria for atypical RTT (7/12), followed by RTT-like (4 cases), while typical RTT seems to be rarer. Microcephaly is present in less than half, and global DD is usually evident since the first months of life, while the remaining patients regress (6–24 months). Patients with atypical/RTT-like features have EOEE or experience neonatal epilepsy, followed by West syndrome. Early-infantile focal seizures (hypertonus followed by eyelids and perioral myoclonic jerks) were also reported [29]. They usually have absent or impaired hand skills and midline, clapping, mouthing, or hand washing stereotypies, and never acquire or have very limited spoken language. Most have impaired gait. The most common supportive criteria in this group include bruxism, impaired sleep, abnormal muscle tone and scoliosis/kyphosis. Additionally reported hyperkinetic MDs include tremors (also head tremor), intentional myoclonus and paroxysmal dyskinesia [13].

### 5.3. Ion Channels

#### 5.3.1. *SCN1A* 

Sodium voltage-gated channel α subunit 1 (*SCN1A*) gene variants are associated with a wide spectrum of conditions in which seizures are a prominent feature. A single patient with an RTT-like phenotype, who is microcephalic, never acquired either language or gait, and did not regress, has been reported in the literature. Epilepsy or HS characteristics were not detailed [36].

#### 5.3.2. *SCN2A* 

Sodium voltage-gated channel α subunit 2 (*SCN2A*) causes various neurodevelopmental phenotypes including DD with hypotonia and autistic features, and DE/EE (OS, EIFMS), depending on the functional effects of genetic variants. Patients with associated MD have been repeatedly described [60]. One case with atypical RTT (showing regression simultaneously to EE onset at 17 months) [2] and one with RTT-like (with infantile-onset EE, severe DD, no functional hand use and stereotypies) [43] have been reported.

#### 5.3.3. *SCN8A* 

One patient with infantile spasms beginning at 4 months and evolving into LGS, experiencing regression on a background of pre-existing DD, received an RTT-like diagnosis based on impaired language and gait, stereotypies and 10/11 supportive criteria [27].

#### 5.3.4. *KCNB1* 

*KCNB1*-related encephalopathy is a DE/EE featuring DD with language impairment, autistic features, dyspraxia (especially oro-motor) and generalized or focal seizures. Stereotypic hand movements have been repeatedly described [61,62], together with other hyperkinetic MDs, including chorea and myoclonus [62]. Apart from one case with typical RTT [2], two patients received a diagnosis of atypical RTT, based on no regression, loss of hand skills and language and impaired gait in one. Both patients had bruxism, abnormal tone and intense eye communication [41].

#### 5.3.5. *KCNQ2* 

Potassium channel, voltage-gated, kqt-like subfamily, member 2 (*KCNQ2*) pathogenic variants cause benign neonatal epilepsy and *KCNQ2*-related encephalopathy. A hyperkinetic MD has been increasingly recognized, but also parkinsonism (bradykinesia, hypomimia and hypoactivity) has been reported [44]. Together with early-onset epilepsy, six patients (three males) also presented with an RTT-like phenotype, featuring regression, abnormal early development, impaired/absent hand use and stereotypies (including hand flapping/washing/mouthing) in half, while the majority are non-ambulant and non-verbal, with profound DD. The most represented (100%) supportive criteria are abnormal tone; only one patient has more than 5 supportive criteria. Of note, two out of six have abnormal brain MRI, including fronto-insular atrophy [12,42,44,45].

#### 5.3.6. *HCN1* 

Hyperpolarization-activated, cyclic nucleotide–gated, 1 (*HCN1*) gene variants are known to cause a spectrum of conditions ranging from genetic epilepsies with febrile seizures plus to DE-EE. One microcephalic patient who never acquired functional hand use or language, lost independent walking and showed HS aged >10 years has been described as showing an RTT-like phenotype, with 2/11 supportive criteria (breathing abnormalities and hypotonia) [63].

### 5.4. Receptors

#### 5.4.1. *GABRB3* 

GABA-A receptor, b3 subunit (*GABRB3*) is associated with focal epilepsy and EE in infancy (onset: birth-15 months). Regression and stagnation can occur over a long time span (3 months–5 years). Autistic features, self-injurious behavior and hyperventilation occur in single patients. One case with focal epilepsy, hand stereotypies, autistic features and an RTT-like phenotype has been reported, although clinical information is limited. Ataxia is relatively frequent, but tremor and dyskinesia have also been reported. Brain MRI is most frequently normal; nevertheless, hypomyelination can be a feature [46].

#### 5.4.2. *GABRG2* 

A single patient with a de novo mosaic pathogenic variant in the GABA-A receptor, γ2 subunit (*GABRG2*) gene with focal myoclonic seizures at 2 months, regression at 12 months, loss of acquired hand movements, HS, impaired ambulation and never acquiring language (no microcephaly) was documented, with 10/11 supportive criteria [13].

#### 5.4.3. *GRIA2* 

Pathogenic variants in the glutamate Ionotropic Receptor AMPA Type Subunit 2 (*GRIA2*) cause a complex neurodevelopmental disorder in which, following a period of normal development, early-onset epilepsy/EE with microcephaly, hypotonia, DD with absent speech, ASD or repetitive behavior and a wide range of MDs, mainly hyperkinetic (dystonia, dyskinesia, startle, chorea, oculogyric crises, stereotypies) but also—less frequently—hypokinetic MD. Between 2 and 6 years, several patients were reported to develop RTT-like features, including HS, screaming episodes, gait abnormalities (ataxia, apraxia), sleep disturbances and an abnormal breathing pattern with hyperventilation episodes. Additional stereotypic movements include head nodding and rocking. Brain MRI is usually abnormal (cerebral/cerebellar atrophy, hypomyelinating leukodytrophy). One girl with IS at 15 months, followed by focal impaired awareness and tonic-clonic seizures was described, with 2/11 supportive Neul’s criteria [47].

#### 5.4.4. *GRIN1* 

Glutamate Ionotropic Receptor NMDA Type Subunit 1 (*GRIN1*) gene causes an EE (EOEE, WS) with background EEG deterioration, micro/macrocephaly, global DD, central visual impairment and a complex MD (oculogyric crises, choreoathethosis, chorea, dystonia, oculo-motor apraxia), with stereotypies (hand but also limbs) [25,64]. Three cases with RTT-like diagnosis have been described. They share infantile-onset epilepsy with abnormal early development, hand apraxia (associated with hand-washing, wringing and mouthing in 2), abnormal breathing and inappropriate laughter/crying in all, while single patients have awake bruxism, disrupted sleep or growth retardation [12,25]. Brain MRI findings can include cerebral and cerebellar atrophy, white matter changes and a thin corpus callosum [25,64].

#### 5.4.5. *GRIN2B* 

Glutamate Ionotropic Receptor NMDA Type Subunit 2B (*GRIN2B*) pathogenic variants are known to cause DEE27, characterized by developmental delay, early-onset seizures, possibly hypotonia, autistic features and autism spectrum disorders. They can also cause an autosomal dominant form of ID (MR AD 6).

Two “RTT-like” patients have been reported, with no regression, although one experienced stagnation at 8 months. They lost ambulation, never acquired language and showed HS, which notably developed after 10 years of age in one. No supportive criteria for RTT were present [36,48].

### 5.5. Transporters

#### 5.5.1. *SLC6A1* 

GAT-1, encoded by Solute Carrier Family 6 Member 1 (*SLC6A1*), is one of the γ-aminobutyric acid (GABA) transporters in the brain, responsible for GABA reuptake from synapses. Its pathogenic variants cause an EE with myoclonic-atonic seizures [65]. Two patients were reported, with an atypical RTT [26] and RTT-like phenotype, respectively [36]. Nor the epilepsy phenotype nor HS have been described.

#### 5.5.2. *SLC35A2* 

The Solute Carrier Family 35 Member A2 (*SLC35A2*) gene causes a congenital disorder of glycosylation, but transferrins isoelectric focusing may be normal after infancy [66]. Patients present mainly with drug-resistant EE with hypsarrhythmia, facial dysmorphism, severe ID, skeletal abnormalities, congenital cardiac disease and cortical visual impairment. One microcephalic, RTT-like patient (stagnating at 6 months) has been documented. He lost functional hand use, had HS, gait abnormalities and impaired language, and 3/11 supportive criteria (impaired sleep, abnormal muscle tone and inappropriate screaming spells). Brain MRI was abnormal (thin corpus callosum, brain atrophy and periventricular heterotopias) [11].

### 5.6. Transcription Factors

#### 5.6.1. *MEF2C* 

Patients with myocyte enhancer factor 2 (*MEF2C*) gene variants present with cognitive impairment, gross motor delay, speech disorder and autistic features, in a proportion of cases with associated drug-responsive epilepsy, usually starting between 6 and 18 months of age. Although some show a phenotype suggestive of the “early-onset seizures” variant (severe hypotonia and ID, absent speech, epilepsy and stereotypies), although without regression [49,50]; in the majority of cases HS (hand mouthing and washing) [50] are the only suggestive clinical sign for RTT [67], and purposeful hand use is generally retained. Aside from one patient with typical RTT [50], data from eight RTT-like patients show hand clasping, wringing or mouthing in all, a low rate of supportive criteria in the majority, and frequently abnormal brain MRI [42,49,50,51].

#### 5.6.2. *ACTL6B* 

A single RTT-like patient harboring an actin-like protein 6b (*ACTL6B*) gene variant with EE (IS at 3 months), followed by bilateral tonic-clonic seizures and generalized cerebral atrophy, with severe DD (non-verbal and non-ambulant) and hand wringing stereotypies but no history of regression was reported [52].

### 5.7. Axon Guidance

#### *NTNG1* 

Two patients harboring Netrin G1 (*NTNG1*) variants were described with atypical RTT (regression, hand apraxia, dyspraxic gait and HS, and unspecified epilepsy since 6 years of age) [26] and with an RTT-like phenotype, respectively. This second patient showed severe DD (non-verbal, wide-based unsteady gait), but no regression, and had head growth deceleration and hand clasping, mouthing and wringing since 2 years of age [54]. This second patient also had minor dysmorphisms, and brain MRI was abnormal (atrophy since infancy, myelination delay since 2 years) [54,68].

### 5.8. Ubiquitination

#### 5.8.1. *RHOBTB2* 

Rho Related BTB Domain Containing 2 (*RHOBTB2*) pathogenic variants cause a DE/EE with a mean age of onset of 5 months, frequent status epilepticus, hypotonia, possible microcephaly, moderate-to-profound ID, possible regression or stagnation and episodes of acute encephalopathy with hemiplegia acutely accompanied by cerebral swelling followed by atrophy. A complex MD (chorea, dystonia, stereotypies, paroxysmal dyskinesia) is also typical [69], with anecdotal response to carbamazepine [70]. A single patient fulfilled criteria for an RTT-like diagnosis, having 8/11 supportive criteria [11].

#### 5.8.2. *HECW2* 

Pathogenic variants in the HECT, C2 and WW domains-containing E3 ubiquitin-protein ligase 2 (*HECW2*) gene, encoding a member of the E3 ubiquitin ligase family stabilizing p73, cause ID, drug-resistant epilepsy, central visual impairment and frequently cortical and cerebellar atrophy with a thin corpus callosum. One patient with tonic epilepsy since 3 years of age and dysmorphic features had an RTT-like phenotype (5/11 supportive criteria), with HS (tapping, flapping and wringing not at the midline). Notably, she had late-onset regression at 12 years and did not show microcephaly [53].

### 5.9. Intellectual Disability and Epilepsy Genes

#### 5.9.1. Synapsis

##### *IQSEC2* 

IQ motif and Sec7 domain 2 (*IQSEC2*) is a cause of X-linked ID with seizures. It encodes for a guanine nucleotide exchange factor. Additional features include autistic features, dysmorphisms and brachi- and plagiocephaly. Overlapping clinical features between *IQSEC2*-related disorder, RTT and PHS have been described. Twenty-one patients with ID, epilepsy and RTT-like features share regression in nearly half (although EE-related in one, involving language in one and of late occurrence in one, >5 years of age), frequent DD since infancy, impaired gait (mostly ataxic) and the universal occurrence of stereotypies not limited to hands (flapping, shaking, wringing, “wiping”) but also including teeth gnashing, bruxism, head banging and shaking, and rocking. Brain MRI is usually abnormal (mainly atrophy and white matter changes) [55,56,57,58,59].

#### 5.9.2. Transcription Regulation/Modification

##### *HNRNPH2* 

Pathogenic variants in the heterogeneous nuclear ribonucleoprotein H2 (*HNRNPH2*) gene cause “Bain type” syndromic X-linked ID, with possible coexistence of epilepsy. Stereotypies can be present, not necessarily in the context of autism. Brain MRI can be significant for cerebellar vermis hypoplasia and subcortical atrophy [71,72]. Severe ID with abnormal tone, absent speech and ataxic gait can suggest RTT, although the main clinical features are absent, including regression (documented once, at 20 years of age, in a patient without epilepsy), and abnormal development is detectable since the first months of life. However, during disease course, the association of stereotypies with breathing and sleep disruption can lead to a diagnosis of an RTT-like phenotype. Of note, adult-onset oro-mandibular dystonia (at 19 years) and tremor (since age 31 years) have been described [72].

#### *EEF1A2* 

One patient with epilepsy since 1 month of age, no regression and no microcephaly received a RTT-like diagnosis due to the presence of HS and 8/11 supportive criteria (breathing and sleep abnormalities, awake bruxism, laughter and screaming spells, abnormal tone, kyphoscoliosis, growth retardation and reduced sensitivity to pain) was described harboring a gene variant in the Eukaryotic translation elongation factor 1 α 2 (*EEF1A2*) gene [11].

#### 5.9.3. Neurodegenerative Disorders

##### *WDR45* 

The WD repeat domain 45 (*WDR45*) gene, involved in autophagy, causes neurodegeneration with brain iron accumulation (NBIA), a disease characterized by global DD in childhood, followed by regression in early adulthood (progressive dystonia, parkinsonism and dementia). Brain MRI shows iron accumulation in the substantia nigra (with a ‘halo’ of T1 hyperintensity) and globus pallidus [73].

Overlapping features between its early stages and RSS include DD, HS, epilepsy, sleep impairment and spasticity. Out of the original 23 patients, atypical RTT was suspected in seven (30%), of whom six had epilepsy [73]. A further study confirmed RTT-like features in 20% of cases [74].

Single reports or small case series detailed revised Neul’s criteria in 10 patients (Table S2). One received a clinical diagnosis of typical RTT [25], while the remaining had RTT-like features. Five were microcephalic, and four had regression. The loss of hand use occurred in five, loss of speech in two, and abnormal gait in six. More than five supportive criteria were present just in the typical RTT case [25]. The most frequent finding was bruxism (7/10), followed by abnormal tone (6/10), inappropriate laughing/screaming spells (5/10) and intense eye communication (4/10) [25,41,75,76,77,78,79].

HS (present in 8/10, with onset—when reported—between 6 months and 4 years) include mouthing, wringing, washing, rubbing, licking or finger-sucking. However, additional stereotypies are possible, including body rocking, flicking things with fingers [76] and lip smacking [77], in some cases in the context of diagnosed autism spectrum disorder (ASD) [76].

#### 5.9.4. *PPT1* 

Although one Ceroidolipofuscinosis type 1 (CLN1) (caused by pathogenic variants in the palmitoyl-protein thioesterase 1-*PPT1*-gene) patient was reported as mimicking RTT because of HS during follow-up, onset was marked by motor impairment [80]. A further case with motor, speech and cognitive regression at 17 months and concomitant onset of HS had further deterioration with the loss of ambulation and continence at 3 years and epilepsy and global cerebral atrophy since 4 years. Six supportive criteria for RTT were present (abnormal breathing, bruxism, impaired sleep, abnormal tone, growth retardation and reduced sensitivity to pain) [12] (Table S2).

#### *MFSD8* 

CLN7 is an autosomal recessive disorder caused by pathogenic variants in the Major Facilitator Superfamily Domain Containing 8 (*MFSD8*) gene [81]. Phenotypes are rather homogeneous, with symptoms onset between 2 and 11 years (mean: 5 years) [82]. Two cases presenting with RTT-like features have been described, exhibiting normal development until 12 months [82] and 2.5 years [81], when regression occurred. Of note, in one case, apparent (transient) stabilization ensued [80], while the second had partial language recovery [83]. Both patients satisfied RTT major criteria and had HS (clapping and mouthing) since 18 months [82] and 3.5 years [83], respectively. Subsequent disease course was overtly neurodegenerative [82,83]. Brain MRI showed atrophy and leucopathy at 5 years. Among supportive criteria, only abnormal tone (in both) and abnormal breathing (in one) were present [82] (Table S2).

##### *EIF2B2* 

Language and hand manipulation regressed at 24 months in one patient, followed by autistic-like behavior since 30 months; concomitantly HS, ataxia, rigidity and screaming spells emerged, along with continuous tremor. She had epilepsy (no information provided). Brain MRI was normal at 3 years. The patient fulfilled 3/4 major criteria, had no exclusion criteria and showed 5/11 supportive criteria (abnormal tone, vasomotor disturbances, scoliosis, small cold hands and feet, screaming spells) (Table S2) [11]. Biallelic pathogenic variants in the Eukaryotic Translation Initiation Factor 2B Subunit β (*EIF2B2*) are the cause of leukoencephalopathy with vanishing white matter.

There are additional, anecdotal descriptions of neurodegenerative conditions with epilepsy and RTT-like features, although not associated in single patients (i.e., ST3 β-Galactoside α-2,3-Sialyltransferase 5, *ST3GAL5* gene) [84].

## 6. Epilepsy and DE/EE Genes with Stereotypies

Finally, stereotypies can be part of complex neurological phenotypes including DD and often a spectrum of different MDs outside the RSS. Aside from previously discussed genes, additional monogenic causes, with or without ASD or autistic features, should be considered: adenylosuccinate lyase deficiency [85,86], *GABRA1* (γ-aminobutyric acid type A receptor alpha1 subunit) [87], *KCNQ3* (Potassium Voltage-Gated Channel Subfamily Q Member 3) [88], *PCDH19* (Protocadherin 19) [89], *SETD5* (SET Domain Containing 5) and *TBL1XR1* (TBL1X Receptor 1) [90], *AP3B2* (Adaptor-Related Protein Complex 3 β 2 Subunit) [91], *CACNA1B* (Calcium Voltage-Gated Channel Subunit Alpha1 B) [92], *GNAO1* [93] and *SZT2* (seizure threshold 2) related disorders [94].

## 7. Clinical Remarks

We reviewed the main groups of monogenic conditions with DE-EE and published cases clinically labeled as falling within the RSS, for whom clinical description allowed the application of Neul’s criteria. We aimed to investigate the clinical features associated with the use of the “Rett-like” label. Although the diagnosis of complex NDDs necessarily relies on NGS technologies (either targeted gene panels or whole exome sequencing) [95], some final remarks might help correct results interpretation in the clinical setting through accurate phenotyping.

Reviewing these DE-EE patients’ characteristics in light of Neul’s criteria shows that different disorders and pathogenic mechanisms can lead to partially overlapping clinical features. First of all, developmental regression is described either in single reports or in half (*KCNQ2*, *SLC6A1* and *NTNG1*) or less (*STXBP1*, *KCNB1*, *GRIN1*, *IQSEC2*) of cases with multiple described patients. Age at regression is reported in a few papers, and late occurrence is exceptional (i.e., 5 and 9 years in *IQSEC2*; 12 years in *HECW2*) [1]. This, together with the *HDAC8*-positive CdL case with regression at 24 months followed by further deterioration at 6 years, highlights an important difference with typical RTT, in which regression, followed by stabilization, occurs within the first year of life and is followed by further deterioration only in late teens/early adulthood. Importantly, regression can occur at EE onset (*SCN2A*, *SCN8A* or some *IQSEC2* cases [58]) but also before (*KCNA2*, *KCNB1*, *GARBRB2* and *MEF2C*) or after (*STXBP1*, *GARBRG2*, *HECW2* and *GRIN1*), suggesting different underlying pathomechanisms (EE-related or mainly driven by the genetic condition itself) [56]. In other patients, stagnation occurs, either at the same age as DE onset (*RHOBTB2* and *SLC35A2*) or before epilepsy onset (*GRIN2B*).

The loss of purposeful hand movements is described in 15–67% of patients in disorders with multiple descriptions. However, never acquired/impaired hand skills without regression also seem to be common, especially in *STXBP1* (7/13 patients), *KCNQ2* (3/6), *GRIN2B*, *HNRNPU* and *SLC6A1* (1/2) and are also reported in single cases harboring *GRIN1* or *HCN1* variants. Similarly, although language regression is uncommon, its impairment or absence are described in *STXBP1* (7/13 patients), *KCNQ2* (5/6), *MEF2C* (7/9), *SCN2A, HNRNPU* and *GRIN2B* (2/2), *KCNB1* (1/3) and, in single reports, (*KCNA2*, *GABRG2*, *SCN1A*). Therefore, the “ultimate” phenotype might appear more homogeneously reminiscent of RTT syndrome, but patients’ natural history can significantly diverge from RTT (Table S1, Figure 1 and Figure 2).

The partial/complete loss of ambulation is uncommon, occurring in *STXBP1* (1/13 patients), *IQSEC2* (1/12), *GRIN2B* (2/2) and *HCN1* (1/1). In ambulatory patients, gait patterns are described in a subset of cases, with three main patterns: ataxia or wide-based (*STXBP1*: 4/13 patients; *IQSEC2*: 4/8; *MEF2C*: 3/9; *NTNG1*: 1/2 and *HNRNPH2*: 1/1); dyspraxic (*NTNG1*: 1/2; *GABBR2*, *HDAC4*, *ANXA11*, *KIF4B*, *RRN3*, and *JMJD1C*: 1/1) and ataxic-dyspraxic gait (*STXBP1*: 1/13; *KCNQ2*: 1/6).

Stereotypies characteristics were detailed in a minority of cases (Table S1) and are more often multiple. They include midline, clasping, mouthing and hand washing (*STXBP1*), rubbing and hand mouthing (*GABBR2*), hand wringing (*KCNB*, *ACTL6B*), clasping, mouthing and wringing (*NTNG1*), hand clapping, wringing, and mouthing (*MEF2C*), hand tapping, flapping and wringing (*HECW2*), hand flapping, washing and rubbing, and fits to midline (*IQSEC2*), hand washing (*JMJD1C*) and clapping (*HNRNPU*). Therefore, HS can diverge from typical hand stereotypies to the midline or combine with non-RTT-specific stereotypies, such as head nodding and rocking (*GRIA2*), tongue-protruding movements (*IQSEC2*) or undetermined “stereotypic movements” (*KCNQ2*), underscoring the coexistence of features typical or reminiscent of RSS with presumably non-RTT-related MD, which might in some instances be ASD- or DD-related.

Moreover, the frequency of additional supportive criteria and their distribution vary widely, with only abnormal tone and impaired gait involving a high number of cases, contributing to further phenotypic heterogeneity (Table S1, Figure 2). This phenotypic variability probably reflects the absence of diagnostic criteria for “RTT-like”, whose use has spread to describe patients reminiscent of RTT but not fulfilling typical or atypical RTT criteria, and who (in some cases) might be better considered affected by genetic NDDs to be included in the RSS differential. Correctly distinguishing between RTT and its “mimics” might carry management implications, as specific RTT therapies become available [96]. Finally, in some reviewed papers, part of the clinical information is missing, preventing deeper understanding of the full clinical phenotype and further comparison between disorders.

**Figure 3:**
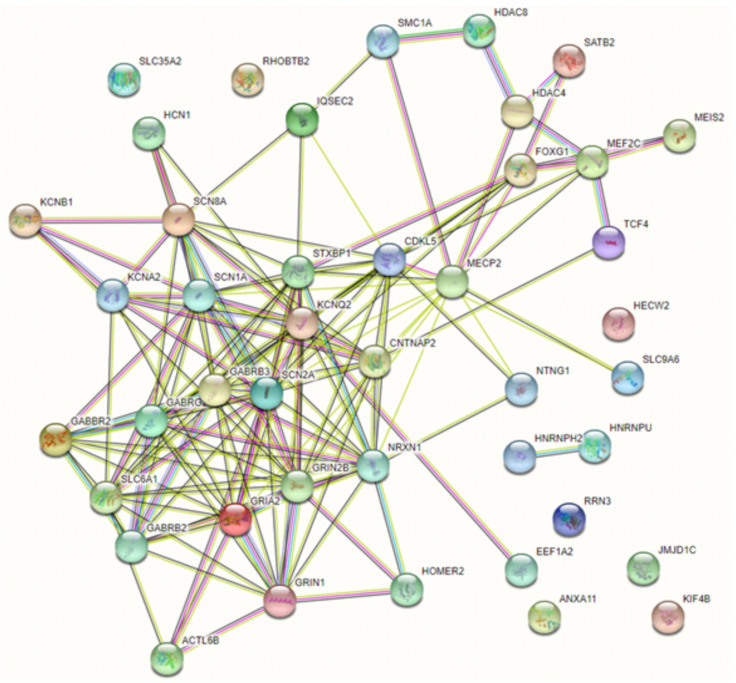
Known functional interaction networks between reviewed genes. A first network is represented by genes causative of Rett, atypical Rett syndrome and some of their most important differentials (Cornelia de Lange syndrome, Pitt–Hopkins and Pitt–Hopkins-like syndrome). A second network is presented by genes causing complex neurodevelopmental disorders in which epilepsy or DE/EE are a cardinal feature, mainly including receptors and ion channels. A third group is represented by genes with determined functional connections only between each other (*HNRNPU* and *HNRNP2*, encoding for RNA-binding proteins) and that currently lack known interactions within these networks.

On the other hand, some of the reviewed genes are functionally related or co-expressed and operate on key cellular functions (chromatin regulators, receptors, ion channels, transporters and synaptic vesicle cycling), suggesting disruption in shared biological pathways. Unsurprisingly, the majority of genes included in this review have multiple known, experimentally determined interactions (and these mainly include the DE-EE genes encoding for ion channels and receptors), while a second group of tightly connected genes involves transcription factors and chromatin modulators. Not unexpectedly, the two members of the heterogeneous nuclear ribonucleoproteins family *HNRNPU* and *HNRNP2* appear functionally related to each other [97] but not with the main network, and additional genes with single RSS case descriptions lack evidence of functional connection or co-expression with the main network (Figure 3). Major alterations affecting several neurological processes, such as the regulation of synaptic transmission, inflammatory and stress-related processes, DNA binding and mRNA stability were confirmed in RTT with a transcriptomic approach [98]. Moreover, the dysregulation of these pathways appears important in other NDD related to RTT, such as epilepsy, AD and intellectual disability [35]. Convergence of cellular pathways can result in a final, common phenotypic core, but careful phenotypic review (i.e., characteristics of stereotypies; age, pathophysiology and domains involved in regression), and molecular data suggest a distinction between “true” RSS and a constellation of (diverse) phenotypic “mimics” [35,36,96], although definite molecular diagnosis is the only way to reliably make a diagnosis [96].

From a diagnostic standpoint, although syndromic conditions are often mainly considered as differential diagnoses versus RSS, the actual description of patients without dysmorphisms/malformations also mandates the consideration of these genes in non-syndromic cases.

Finally, of utmost importance, neurodegenerative disorders can mimic RSS in their early stages, when neuroimaging is normal, and transient stabilization possibly follows regression. From a clinico-diagnostic standpoint, close follow-up is mandatory, and the *WDR45* gene should be added to the genetic work-up in RSS cases.

Based on our literature review, in patients affected by DE/EE with or without regression, in the presence of clinical features reminiscent of RTT but not satisfying Neul’s criteria, we suggest differentiating between syndromic and non-syndromic conditions and excluding neurodegenerative causes. If patients do not show a RTT-like phenotype, but the clinical picture is dominated by a NDD including DE/EE and stereotypies, the range of possible etiologic conditions needs to be widened further, and additional elements of the NDD might prove useful, namely the presence of autistic features.

## 8. Conclusions

Outside typical and atypical RTT, the Rett-like cases we reviewed are marked by high genetic and phenotypic heterogeneity. Patients share a complex neurodevelopmental and neurological phenotype, encompassing DD, epilepsy or DE/EE, and MD with a strong focus on impaired gait, abnormal tone and HS. However, from a clinical standpoint, important differences such as the presence and characteristics of regression, the presence of a period of stabilization and the loss of functional hand use might prove useful for correct diagnostic suspicion but also for deeper insight into different underlying pathomechanisms. In conclusion, in the reviewed cohort, while some Rett features may be present in other disorders, these do not actually seem to be Rett-related.

## Figures and Tables

**Table 1:** Neul’s criteria for typical and atypical Rett syndrome.

Required for Typical RTT	Required for Atypical (Variant) RTT	Main Criteria	Exclusion Criteria for Typical RTT	Supportive Criteria for Atypical RTT
A period of regression followed by recovery or stabilization All main criteria and all exclusion criteria Supportive criteria: not required, although often present	A period of regression followed by recovery or stabilization ≥2 out 4 main criteria 5 out of 11 supportive criteria	Partial/complete loss of acquired purposeful hand skills Partial or complete loss of acquired spoken language Gait abnormalities: impaired (dyspraxic) or absent Stereotypic hand movements (hand wringing, squeezing, clapping, tapping, mouthing and washing/rubbing)	Brain injury secondary to trauma, neurometabolic diseases, or severe infection causing neurological problems Grossly abnormal psychomotor development in the first 6 months of life	Breathing disturbances when awake Bruxism when awake Impaired sleep pattern Abnormal muscle tone Peripheral vasomotor disturbances Scoliosis/kyphosis Growth retardation Small cold hand and feet

**Table 2:** Electroclinical characteristics of typical and atypical RTT syndrome.

Typical RTT	*CDKL5*-Related Disorder	*FOXG1* Syndrome
Clinical Stages	Electroclinical Stages	Epilepsy Features
Early onset phase (6–12 months): loss of acquired motor and language skills and purposeful hand movements Rapid destructive phase (1–3 y): autistic features, intellectual disability, hand stereotypies, abnormal gait/motor dysfunction, onset of abnormal respiratory patterns Stabilization phase (2–10 y): improvements in behavior, eye contact and hand function Late motor deterioration (>10 y): spasticity, dystonia, and scoliosis, loss of independent walking in ambulant patients EPILEPSY FEATURES Mean onset: 4.7 y Frequent FS, No specific seizure semiology	Stage I (Early epilepsy): IS; Tonic-vibratory seizure, followed by a clonic phase with series of spasms, gradually evolving into repetitive distal myoclonic jerks, lasting 2–4 min Onset: neonatal-4th month (median: 4 weeks) Stage II (EE): 6 months–3 years (median: 11 months) IS intermixed with brief tonic seizures profound DD, no language or motor development, massive hypotonia Stage III (Late multifocal and myoclonic epilepsy): ages: 2.5–11 y (median 7 y) drug-resistant epilepsy with tonic seizures and spasms, myoclonic jerks or atypical absences Or epilepsy remission Ages: 2.5–19 y (median 5 y)	Deletions and intragenic variants: epilepsy onset within the second year of life (mean: 22 months in [7]) various epilepsy types (focal impaired awareness, myoclonic, and bilateral tonic) rate of drug resistance is high Duplications: IS (mean age at onset: 7.4 months). Frequent focal seizures (onset: 5 months–6 years), often in association with spasms [8]. in a minority: later recurrence of tonic or myoclonic seizures
EEG	EEG	EEG:
Stage 1: N/posterior rhythms slowing Stage 2: rolandic IED (drowsiness, sleep). sleep architecture abnormalities (poor/absent spindles) Stage 3: abnormal background (posterior slowing, absent sleep figures); bilaterally synchronous bursts of pseudo-periodic delta and generalized rhythmic spikes in sleep Stage 4: abnormal, slow background (wakefulness and sleep), central and/or vertex theta (4–6 Hz), IED (multifocal spikes or sharp waves during wakefulness and generalized slow spike-wave complexes during sleep)	Stage I: Interictal: N/slow. Ictal: bilateral and synchronous flattening, followed by repetitive sharp waves and spikes Stage II: Typical/modified hypsarrhythmia, very slow, intermixed with focal spikes and polyspikes (F, C, O) Stage III: High-amplitude delta with pseudo-periodic bursts of high-amplitude S, PS, SW predominating over the C, T, T-O region	Deletions and intragenic variants: slow background, multifocal S and sharp waves, less frequently diffuse theta excess Duplications: hypsarrhythmia/modified hypsarrhythmia (onset) multifocal S-slowW, bursts of generalized S-slowW, or focal slowing intermixed with high-amplitude irregular S-slowW (follow-up)

**Table 3:** Genes reviewed in this paper, with associated OMIM disorders, inheritance, reported clinical diagnoses within the Rett syndrome spectrum, number of patients and reference. As per OMIM nomenclature, the symbol “#” before a number indicates a descriptive entry (a phenotype), while “*” indicates a gene.

GENE, Function/Name	Associated Disorder/OMIM#	Inheritance	Diagnosis within RSS Spectrum	REF
DE/EE Genes				
Synapsis				
Synaptic Vesicle Cycle				
STXBP1	DEE4 (#612164)	AD	Atypical: 7/12 RTT-like: 4/12 “Typical”: 1/12	[2,9,11,27,29,35]
Ion Channels				
SCN1A	Dravet syndrome (#607208) GEFS+ 2 (#604403)	AD	RTT-like (single case)	[36]
SCN2A	DEE11 (#613721) BFIS3 (#607745)	AD	Atypical: 1/2 RTT-like: 1/2	[2,43]
SCN8A	DEE13(#614558) BFIS5 (#617080) Cognitive impairment w/out cerebellar ataxia (#614306)	AD	RTT-like (single case)	[27]
KCNB1	DEE26 (#616056)	AD	Typical: 1/3 Atypical: 2/3	[2,41]
KCNQ2	DEE7 (#613720) Myokymia (#121200) BNS1 (#121200)	AD	RTT-like: 6/6	[10,42,44,45]
HCN1	DEE24 (#615871) GEFS+10 (#618482)	AD	RTT-like (single case)	[36]
KCNA2	DEE32 (#616366)	AD	Atypical (single case)	[28]
Receptors				
GABRB2	EIEE2 (#617829)	AD	Typical (single case)	[11]
GABRG2	DEE74 (#618396) GEFS+ 3 (#607681) FFS8 (#607681)	AD	Atypical (single case)	[11]
GABRB3	DEE43 (#617113) Susceptibility to CAE, type 5 (#612269)	AD	RTT-like (single case)	[46]
GABBR2	DEE59 (#617904) NDD with poor language and loss of hand skills (#617903)	AD	Atypical (single case)	[26]
GRIA2	NDD with language impairment and behavioral abnormalities (#618917)	AD	RTT-like (single case)	[47]
GRIN1	NDD with/out hyperkinetic movements and seizures, AD (#614254) and AR (#617820)	AD, AR	RTT-like: 3/3	[10,25]
GRIN2B	DEE27 (#616139) MR AD 6 (#613970)	AD	RTT-like: 2/2	[36,48]
Transporters				
SLC6A1	Myoclonic-atonic epilepsy (#616421)	AD	RTT-like: 1/2 Atypical: 1/2	[26,36]
SLC35A2	CDG type II (#300896)	XLD, somatic mosaicism	RTT-like (single case)	[9]
Transcription Factors/Chromatin Modulation Pathways				
MEF2C	MR, stereotypic movements, epilepsy and/or cerebral malformations (#613443)	AD	RTT-like: 8/9 RTT: 1/9	[42,49,50,51]
ACTL6B	DEE76 (#618468) Intellectual developmental disorder with severe speech and ambulation defects (#618470)	ARAD	RTT-like (single case)	[52]
HDAC4	CdLS (#300882)	AD de novo deletion chr2.q37.1-q37.3 (including HDAC4)S	Atypical (single case)	[26]
HDAC8	CdLS (#300882)	XLD	RTT-like (single case)	[33]
MEIS2	Cleft palate, cardiac defects, and MR (#600987)	AD	RTT-like (single case)	[41]
Ubiquitination				
RHOBTB2	DEE64 (#618004)	AD	RTT-like (single case)	[9]
HECW2	NDD with hypotonia, seizures and absent language (#617268)	AD	RTT-like (single case)	[53]
Axon Guidance				
NTNG1	No OMIM disorder (gene number *608818)	AD	Atypical: 1/2 RTT-like: 1/2	[26,54]
ID + E Genes				
IQSEC2	MR, X-linked 1/78 (#309530)	XLD	RTT-like: 21/21	[55,56,57,58,59]
HNRNPH2	MR, X-linked, syndromic, Bain type (#300986)	XLD	RTT-like (single case)	[58]
EEF1A2	DEE33 (#616409) MR, AD 38 (#616393)	AD AD	RTT-like (single case)	[9]
JMJD1C	No OMIM disorder (gene number *604503)	AD	Typical (single case)	[12]
To Be Further Evaluated				
ANXA11	ALS 23 (#617839)	AD	Atypical (single case)	[26]
KIF4B	No OMIM disorder (gene number *609184)	AD	Atypical (single case)	[26]
*RRN3*	No OMIM disorder (gene number *605121)	AD	Atypical (single case)	[26]

## Supplementary Materials

The following are available online at https://www.mdpi.com/article/10.3390/genes12081157/s1, Table S1: Detailed information extracted from reviewed papers on DE/EE-related genes and Rett syndrome spectrum, Table S2: Detailed information extracted from reviewed papers on neurodegenerative conditions with cases of patients presenting with Rett-like features.

## References

[1] Neul J.L., Kaufmann W.E., Glaze D.G., Christodoulou J., Clarke A.J., Bahi-Buisson N., Leonard H., Bailey M.E.S., Schanen N.C., Zappella M. (2010). Rett syndrome: Revised diagnostic criteria and nomenclature. Ann. Neurol..

[2] Schönewolf-Greulich B., Bisgaard A.-M., Møller R., Dunø M., Brøndum-Nielsen K., Kaur S., Van Bergen N., Lunke S., Eggers S., Jespersgaard C. (2018). Clinician’s guide to genes associated with Rett-like phenotypes-Investigation of a Danish cohort and review of the literature. Clin. Genet..

[3] Pintaudi M., Calevo M.G., Vignoli A., Parodi E., Aiello F., Baglietto M.G., Hayek J., Buoni S., Renieri A., Russo S. (2010). Epilepsy in Rett syndrome: Clinical and genetic features. Epilepsy Behav..

[4] Frullanti E., Papa F.T., Grillo E., Clarke A., Ben-Zeev B., Pineda M., Bahi-Buisson N., Bienvenu T., Armstrong J., Martinez A.R. (2019). Analysis of the Phenotypes in the Rett Networked Database. Int. J. Genom..

[5] Nissenkorn A., Levy-Drummer R.S., Bondi O., Renieri A., Villard L., Mari F., Mencarelli M.A., Rizzo C.L., Meloni I., Pineda M. (2015). Epilepsy in Rett syndrome-Lessons from the Rett networked database. Epilepsia.

[6] Hagne I., Witt-Engerstrom I., Hagberg B. (1989). EEG development in Rett syndrome. A study of 30 cases. Electroencephalogr. Clin. Neurophysiol..

[7] Seltzer L.E., Ma M., Ahmed S., Bertrand M., Dobyns W.B., Wheless J., Paciorkowski A.R. (2014). Epilepsy and outcome inFOXG1-related disorders. Epilepsia.

[8] Cellini E., Vignoli A., Pisano T., Falchi M., Molinaro A., Accorsi P., Bontacchio A., Pinelli L., Giordano L., Guerrini R. (2015). The hyperkinetic movement disorder of FOXG1-related epileptic-dyskinetic encephalopathy. Dev. Med. Child Neurol..

[9] Lopes F., Barbosa M., Ameur A., Soares G., de Sá J., Dias A.I., Oliveira G., Cabral P., Temudo T., Calado E. (2016). Identification of novel genetic causes of Rett syndrome-like phenotypes. J. Med. Genet..

[10] Wang J., Zhang Q., Chen Y., Yu S., Wu X., Bao X. (2019). Rett and Rett-like syndrome: Expanding the genetic spectrum to KIF1A and GRIN1 gene. Mol. Genet. Genom. Med..

[11] Cogliati F., Giorgini V., Masciadri M., Bonati M.T., Marchi M., Cracco I., Gentilini D., Peron A., Savini M.N., Spaccini L. (2019). Pathogenic Variants in STXBP1 and in Genes for GABAa Receptor Subunities Cause Atypical Rett/Rett-like Phenotypes. Int. J. Mol. Sci..

[12] Sáez M.A., Fernández-Rodríguez J., Moutinho C., Sanchez-Mut J.V., Gomez A., Vidal E., Petazzi P., Szczesna K., López-Serra P., Lucariello M. (2015). Mutations in JMJD1C are involved in Rett syndrome and intellectual disability. Genet. Med..

[13] Bahi-Buisson N., Nectoux J., Rosas-Vargas H., Milh M., Boddaert N., Girard B., Cances C., Ville D., Afenjar A., Rio M. (2008). Key clinical features to identify girls with CDKL5 mutations. Brain.

[14] Bahi-Buisson N., Kaminska A., Boddaert N., Rio M., Afenjar A., Gérard M., Giuliano F., Motte J., Héron D., Morel M.A.N. (2008). The three stages of epilepsy in patients withCDKL5mutations. Epilepsia.

[15] Guerrini R., Parrini E. (2012). Epilepsy in Rett syndrome, and CDKL5- and FOXG1-gene-related encephalopathies. Epilepsia.

[16] Melani F., Mei D., Pisano T., Savasta S., Franzoni E., Ferrari A.R., Marini C., Guerrini R. (2011). CDKL5 gene-related epileptic encephalopathy: Electroclinical findings in the first year of life. Dev. Med. Child Neurol..

[17] Kortüm F., Das S., Flindt M., Morris-Rosendahl D., Stefanova I., Goldstein A., Horn D., Klopocki E., Kluger G., Martin P. (2011). The core FOXG1 syndrome phenotype consists of postnatal microcephaly, severe mental retardation, absent language, dyskinesia, and corpus callosum hypogenesis. J. Med. Genet..

[18] Vegas N., Cavallin M., Maillard C., Boddaert N., Toulouse J., Schaefer E., Lerman-Sagie T., Lev D., Magalie B., Moutton S. (2018). Delineating FOXG1 syndrome: From congenital microcephaly to hyperkinetic encephalopathy. Neurol. Genet..

[19] Papandreou A., Schneider R.B., Augustine E.F., Ng J., Mankad K., Meyer E., McTague A., Ngoh A., Hemingway C., Robinson R. (2016). Delineation of the movement disorders associated with FOXG1 mutations. Neurology.

[20] Ehrhart F., Sangani N.B., Curfs L.M. (2018). Current developments in the genetics of Rett and Rett-like syndrome. Curr. Opin. Psychiatry.

[21] Temudo T., Santos M., Dias K., Calado E., Carrilho I., Oliveira G.G., Barbot C., Fonseca M., Cabral A., Dias A. (2007). Stereotypies in Rett syndrome: Analysis of 83 patients with and without detected MECP2 mutations. Neurology.

[22] Brunetti-Pierri N., Paciorkowski A.R., Ciccone R., Della Mina E., Bonaglia M.C., Borgatti R., Schaaf C.P., Sutton V.R., Xia Z., Jelluma N. (2010). Duplications of FOXG1 in 14q12 are associated with developmental epilepsy, mental retardation, and severe speech impairment. Eur. J. Hum. Genet..

[23] Milh M., Villeneuve N., Chouchane M., Kaminska A., Laroche C., Barthez M.A., Gitiaux C., Bartoli C., Borges-Correia A., Cacciagli P. (2011). Epileptic and nonepileptic features in patients with early onset epileptic encephalopathy and STXBP1 mutations. Epilepsia.

[24] Larsen J., Carvill G.L., Gardella E., Kluger G., Schmiedel G., Barisic N., Depienne C., Brilstra E., Mang Y., Nielsen J.E.K. (2015). The phenotypic spectrum of SCN8A encephalopathy. Neurology.

[25] Ohba C., Kato M., Takahashi S., Lerman-Sagie T., Lev D., Terashima H., Kubota M., Kawawaki H., Matsufuji M., Kojima Y. (2014). Early onset epileptic encephalopathy caused by de novo SCN8A mutations. Epilepsia.

[26] Yoo Y., Jung J., Lee Y.-N., Lee Y., Cho H., Na Bs E., Hong J., Kim E., Lee J.S., Lee J.S. (2017). GABBR2mutations determine phenotype in rett syndrome and epileptic encephalopathy. Ann. Neurol..

[27] Olson H.E., Tambunan D., LaCoursiere C., Goldenberg M., Pinsky R., Martin E., Ho E., Khwaja O., Kaufmann W.E., Poduri A. (2015). Mutations in epilepsy and intellectual disability genes in patients with features of Rett syndrome. Am. J. Med. Genet. A.

[28] Allou L., Julia S., Amsallem D., El Chehadeh S., Lambert L., Thevenon J., Duffourd Y., Saunier A., Bouquet P., Pere S. (2016). Rett-like phenotypes: Expanding the genetic heterogeneity to the KCNA2 gene and first familial case of CDKL5-related disease. Clin. Genet..

[29] Romaniello R., Saettini F., Panzeri E., Arrigoni F., Bassi M.T., Borgatti R. (2015). A de-novo STXBP1 gene mutation in a patient showing the Rett syndrome phenotype. NeuroReport.

[30] Huisman S., Mulder P.A., Redeker E., Bader I., Bisgaard A.-M., Brooks A., Cereda A., Cinca C., Clark D., Cormier-Daire V. (2017). Phenotypes and genotypes in individuals with SMC1A variants. Am. J. Med. Genet. A.

[31] Lebrun N., Lebon S., Jeannet P.-Y., Jacquemont S., Billuart P., Bienvenu T. (2015). Early-onset encephalopathy with epilepsy associated with a novel splice site mutation inSMC1A. Am. J. Med. Genet. A.

[32] Goldstein J.H., Tim-Aroon T., Shieh J., Merrill M., Deeb K.K., Zhang S., Bass N.E., Bedoyan J.K. (2015). Novel SMC1A frameshift mutations in children with developmental delay and epilepsy. Eur. J. Med. Genet..

[33] Saikusa T., Hara M., Iwama K., Yuge K., Ohba C., Okada J.-I., Hisano T., Yamashita Y., Okamoto N., Saitsu H. (2018). De novo HDAC8 mutation causes Rett-related disorder with distinctive facial features and multiple congenital anomalies. Brain Dev..

[34] Hara M., Ohba C., Yamashita Y., Saitsu H., Matsumoto N., Matsuishi T. (2015). De novoSHANK3mutation causes Rett syndrome-like phenotype in a female patient. Am. J. Med. Genet. A.

[35] Sajan S.A., Jhangiani S.N., Muzny D.M., Gibbs R.A., Lupski J.R., Glaze D.G., Kaufmann W.E., Skinner S.A., Annese F., Friez M.J. (2016). Enrichment of mutations in chromatin regulators in people with Rett syndrome lacking mutations in MECP2. Genet. Med..

[36] Lucariello M., Vidal E., Vidal S., Saez M., Roa L., Huertas D., Pineda M., Dalfó E., Dopazo J., Jurado P. (2016). Whole exome sequencing of Rett syndrome-like patients reveals the mutational diversity of the clinical phenotype. Qual. Life Res..

[37] Lee J.S., Yoo Y., Lim B.C., Kim K.J., Choi M., Chae J. (2016). SATB2-associated syndrome presenting with Rett-like phenotypes. Clin. Genet..

[38] LeDuc M.S., Chao H.-T., Qu C., Walkiewicz M., Xiao R., Magoulas P., Pan S., Beuten J., He W., Bernstein J.A. (2017). Clinical and molecular characterization of de novo loss of function variants inHNRNPU. Am. J. Med. Genet. A.

[39] Shimada S., Oguni H., Otani Y., Nishikawa A., Ito S., Eto K., Nakazawa T., Yamamoto-Shimojima K., Takanashi J.-I., Nagata S. (2018). An episode of acute encephalopathy with biphasic seizures and late reduced diffusion followed by hemiplegia and intractable epilepsy observed in a patient with a novel frameshift mutation in HNRNPU. Brain Dev..

[40] Spagnoli C., Rizzi S., Salerno G.G., Frattini D., Koskenvuo J., Fusco C. (2021). Pharmacological Treatment of Severe Breathing Abnormalities in a Case of HNRNPU Epileptic Encephalopathy. Mol. Syndromol..

[41] Srivastava S., Desai S., Cohen J., Smith-Hicks C., Barañano K., Fatemi A., Naidu S. (2018). Monogenic disorders that mimic the phenotype of Rett syndrome. Neurogenetics.

[42] Vidal S., Xiol C., Pascual-Alonso A., O’Callaghan M., Pineda M., Armstrong J. (2019). Genetic Landscape of Rett Syndrome Spectrum: Improvements and Challenges. Int. J. Mol. Sci..

[43] Liang J.-S., Lin L.-J., Yang M.-T., Wang J.-S., Lu J.-F. (2017). The therapeutic implication of a novel SCN2A mutation associated early-onset epileptic encephalopathy with Rett-like features. Brain Dev..

[44] Mastrangelo M., Manti F., Giannini M.T., Guerrini R., Leuzzi V. (2020). KCNQ2 encephalopathy manifesting with Rett-like features: A follow-up into adulthood. Neurol. Genet..

[45] Spagnoli C., Salerno G.G., Frattini D., Fusco C. (2018). Two Cases of KCNQ2 Encephalopathy with Unusual Findings: Clinical and Neurophysiological Follow-Up. Clin. Pediatr..

[46] Møller R.S., Wuttke T.V., Helbig I., Marini C., Johannesen K.M., Brilstra E.H., Vaher U., Borggraefe I., Talvik I., Talvik T. (2017). Mutations in GABRB3: From febrile seizures to epileptic encephalopathies. Neurology.

[47] Salpietro V., Dixon C.L., Guo H., Bello O.D., Vandrovcova J., Efthymiou S., Maroofian R., Heimer G., Burglen L., SYNAPS Study Group (2019). AMPA receptor GluA2 subunit defects are a cause of neurodevelopmental disorders. Nat. Commun..

[48] Kyriakopoulos P., McNiven V., Carter M.T., Humphreys P., Dyment D., Fantaneanu T.A. (2018). Atypical Rett Syndrome and Intractable Epilepsy with Novel GRIN2B Mutation. Child Neurol. Open.

[49] Bienvenu T., Diebold B., Chelly J., Isidor B. (2012). Refining the phenotype associated with MEF2C point mutations. Neurogenetics.

[50] Wang J., Zhang Q., Chen Y., Yu S., Wu X., Bao X., Wen Y. (2018). Novel MEF2C point mutations in Chinese patients with Rett (−like) syndrome or non-syndromic intellectual disability: Insights into genotype-phenotype correlation. BMC Med. Genet..

[51] Rocha H., Sampaio M., Rocha R., Fernandes S., Leão M. (2016). MEF2C haploinsufficiency syndrome: Report of a new MEF2C mutation and review. Eur. J. Med. Genet..

[52] Bell S., Rousseau J., Peng H., Aouabed Z., Priam P., Theroux J.-F., Jefri M., Tanti A., Wu H., Kolobova I. (2019). Mutations in ACTL6B Cause Neurodevelopmental Deficits and Epilepsy and Lead to Loss of Dendrites in Human Neurons. Am. J. Hum. Genet..

[53] Nakamura H., Uematsu M., Numata-Uematsu Y., Abe Y., Endo W., Kikuchi A., Takezawa Y., Funayama R., Shirota M., Nakayama K. (2018). Rett-like features and cortical visual impairment in a Japanese patient with HECW2 mutation. Brain Dev..

[54] Borg I., Freude K., Kübart S., Hoffmann K., Menzel C., Laccone F., Firth H.V., A Ferguson-Smith M., Tommerup N., Ropers H.-H. (2005). Disruption of Netrin G1 by a balanced chromosome translocation in a girl with Rett syndrome. Eur. J. Hum. Genet..

[55] Gandomi S.K., Gonzalez K.D.F., Parra M., Shahmirzadi L., Mancuso J., Pichurin P., Temme R., Dugan S., Zeng W., Tang S. (2013). Diagnostic Exome Sequencing Identifies Two Novel IQSEC2 Mutations Associated with X-Linked Intellectual Disability with Seizures: Implications for Genetic Counseling and Clinical Diagnosis. J. Genet. Couns..

[56] Radley J.A., O’Sullivan R.B., Turton S.E., Cox H., Vogt J., Morton J., Jones E., Smithson S., Lachlan K., Rankin J. (2019). Deep phenotyping of 14 new patients with IQSEC2 variants, including monozygotic twins of discordant phenotype. Clin. Genet..

[57] Mau-Them F.T., Willems M., Albrecht B., Sanchez E., Puechberty J., Endele S., Schneider A., Pallares N.R., Missirian C., Rivier F. (2013). Expanding the phenotype of IQSEC2 mutations: Truncating mutations in severe intellectual disability. Eur. J. Hum. Genet..

[58] Lopergolo D., Privitera F., Castello G., Lo Rizzo C., Mencarelli M.A., Pinto A.M., Ariani F., Currò A., Lamacchia V., Canitano R. (2021). IQSEC2 disorder: A new disease entity or a Rett spectrum continuum?. Clin. Genet..

[59] Barrie E.S., Cottrell C.E., Gastier-Foster J., Hickey S.E., Patel A.D., Santoro S.L., Alfaro M.P. (2020). Genotype-phenotype correlation: Inheritance and variant-type infer pathogenicity in IQSEC2 gene. Eur. J. Med. Genet..

[60] Howell K.B., McMahon J.M., Carvill G.L., Tambunan D., Mackay M.T., Rodriguez-Casero V., Webster R., Clark D., Freeman J.L., Calvert S. (2015). SCN2A encephalopathy: A major cause of epilepsy of infancy with migrating focal seizures. Neurology.

[61] Marini C., Romoli M., Parrini E., Costa C., Mei D., Mari F., Parmeggiani L., Procopio E., Metitieri T., Cellini E. (2017). Clinical features and outcome of 6 new patients carrying de novo KCNB1 gene mutations. Neurol. Genet..

[62] Saitsu H., Akita T., Tohyama J., Goldberg-Stern H., Kobayashi Y., Cohen R., Kato M., Ohba C., Miyatake S., Tsurusaki Y. (2015). De novo KCNB1 mutations in infantile epilepsy inhibit repetitive neuronal firing. Sci. Rep..

[63] Nava C., Dalle C., Rastetter A., Striano P., De Kovel C.G.F., Nabbout R., Cancès C., Ville D., Brilstra E.H., EuroEPINOMICS RES Consortium (2014). De novo mutations in HCN1 cause early infantile epileptic encephalopathy. Nat. Genet..

[64] Pironti E., Granata F., Cucinotta F., Gagliano A., Efthymiou S., Houlden H., Salpietro V., Di Rosa G. (2018). Electroclinical history of a five-year-old girl with GRIN1-related early-onset epileptic encephalopathy: A video-case study. Epileptic Disord..

[65] Carvill G.L., McMahon J.M., Schneider A., Zemel M., Myers C.T., Saykally J., Nguyen J., Robbiano A., Zara F., Specchio N. (2015). Mutations in the GABA Transporter SLC6A1 Cause Epilepsy with Myoclonic-Atonic Seizures. Am. J. Hum. Genet..

[66] Yates T.M., Suri M., Desurkar A., Lesca G., Wallgren-Pettersson C., Hammer T.B., Raghavan A., Poulat A.-L., Møller R., Thuresson A.-C. (2018). SLC35A2-related congenital disorder of glycosylation: Defining the phenotype. Eur. J. Paediatr. Neurol..

[67] Lambert L., Bienvenu T., Allou L., Valduga M., Echenne B., Diebold B., Mignot C., Héron D., Roth V., Saunier A. (2012). MEF2C mutations are a rare cause of Rett or severe Rett-like encephalopathies. Clin. Genet..

[68] Nectoux J., Girard B., Bahi-Buisson N., Prieur F., Afenjar A., Rosas-Vargas H., Chelly J., Bienvenu T. (2007). Netrin G1 Mutations Are an Uncommon Cause of Atypical Rett Syndrome with or Without Epilepsy. Pediatr. Neurol..

[69] Straub J., Konrad E.D., Grüner J., Toutain A., Bok L.A., Cho M.T., Crawford H.P., Dubbs H., Douglas G., Jobling R. (2018). Missense Variants in RHOBTB2 Cause a Developmental and Epileptic Encephalopathy in Humans, and Altered Levels Cause Neurological Defects in Drosophila. Am. J. Hum. Genet..

[70] Spagnoli C., Soliani L., Caraffi S.G., Baga M., Rizzi S., Salerno G.G., Frattini D., Garavelli L., Koskenvuo J., Pisani F. (2020). Paroxysmal movement disorder with response to carbamazepine in a patient with RHOBTB2 developmental and epileptic encephalopathy. Park. Relat. Disord..

[71] Bain J.M., Cho M.T., Telegrafi A., Wilson A., Brooks S., Botti C., Gowans G., Autullo L.A., Krishnamurthy V., Willing M.C. (2016). Variants in HNRNPH2 on the X Chromosome Are Associated with a Neurodevelopmental Disorder in Females. Am. J. Hum. Genet..

[72] Peron A., Novara F., La Briola F., Merati E., Giannusa E., Segalini E., Anniballi G., Vignoli A., Ciccone R., Canevini M.P. (2020). Missense variants in the Arg206 residue of HNRNPH2: Further evidence of causality and expansion of the phenotype. Am. J. Med. Genet. A.

[73] Hayflick S.J., Kruer M.C., Gregory A., Haack T.B., Kurian M.A., Houlden H.H., Anderson J., Boddaert N., Sanford L., Harik S.I. (2013). β-Propeller protein-associated neurodegeneration: A new X-linked dominant disorder with brain iron accumulation. Brain.

[74] Belohlavkova A., Sterbova K., Betzler C., Burkhard S., Panzer A., Wolff M., Lassuthova P., Vlckova M., Kyncl M., Benova B. (2020). Clinical features and blood iron metabolism markers in children with β-propeller protein associated neurodegeneration. Eur. J. Paediatr. Neurol..

[75] Ohba C., Nabatame S., Iijima Y., Nishiyama K., Tsurusaki Y., Nakashima M., Miyake N., Tanaka F., Ozono K., Saitsu H. (2014). De novo WDR45 mutation in a patient showing clinically Rett syndrome with childhood iron deposition in brain. J. Hum. Genet..

[76] Chard M., Appendino J.P., Bello-Espinosa L.E., Curtis C., Rho J.M., Wei X.-C., Al-Hertani W. (2019). Single-center experience with β-propeller protein-associated neurodegeneration (BPAN); expanding the phenotypic spectrum. Mol. Genet. Metab. Rep..

[77] Hoffjan S., Ibisler A., Tschentscher A., Dekomien G., Bidinost C., Rosa A.L. (2016). WDR45 mutations in Rett (-like) syndrome and developmental delay: Case report and an appraisal of the literature. Mol. Cell. Probes.

[78] Okamoto N., Ikeda T., Hasegawa T., Yamamoto Y., Kawato K., Komoto T., Imoto I. (2014). Early manifestations of BPAN in a pediatric patient. Am. J. Med. Genet. A.

[79] Kulikovskaja L., Sarajlija A., Savic-Pavicevic D., Dobricic V., Klein C., Westenberger A. (2018). WDR45 mutations may cause a MECP2 mutation-negative Rett syndrome phenotype. Neurol. Genet..

[80] Topçu M., Tan H., Yalnizoğlu D., Usubutun A., Saatçi I., Aynaci M., Anlar B., Topaloğlu H., Turanli G., Köse G. (2004). Evaluation of 36 patients from Turkey with neuronal ceroid lipofuscinosis: Clinical, neurophysiological, neuroradiological and histopathologic studies. Turk. J. Pediatr..

[81] Pao S.S., Paulsen I.T., Saier M.H. (1998). Major Facilitator Superfamily. Microbiol. Mol. Biol. Rev..

[82] Craiu D., Dragostin O., Dica A., Hoffman-Zacharska D., Gos M., Bastian A.E., Gherghiceanu M., Rolfs A., Nahavandi N., Craiu M. (2015). Rett-like onset in late-infantile neuronal ceroid lipofuscinosis (CLN7) caused by compound heterozygous mutation in the MFSD8 gene and review of the literature data on clinical onset signs. Eur. J. Paediatr. Neurol..

[83] Kozina A.A., Okuneva E.G., Baryshnikova N.V., Krasnenko A.Y., Tsukanov K.Y., Klimchuk O.I., Kondakova O., Larionova A.N., Батышева T.T., Surkova E.I. (2018). A novel MFSD8 mutation in a Russian patient with neuronal ceroid lipofuscinosis type 7: A case report. BMC Med. Genet..

[84] Lee J.S., Yoo Y., Lim B.C., Kim K.J., Song J., Choi M., Chae J.-H. (2016). GM3 synthase deficiency due toST3GAL5variants in two Korean female siblings: Masquerading as Rett syndrome-like phenotype. Am. J. Med. Genet. A.

[85] Jurecka A., Zikanova M., Tylki-Szymanska A., Krijt J., Bogdanska A., Gradowska W., Mullerova K., Sykut-Cegielska J., Kmoch S., Pronicka E. (2008). Clinical, biochemical and molecular findings in seven Polish patients with adenylosuccinate lyase deficiency. Mol. Genet. Metab..

[86] Macchiaiolo M., Buonuomo P.S., Mastrogiorgio G., Bordi M., Testa M.B.C., Weber G., Bellacchio E., Tartaglia M., Cecconi F., Bartuli A. (2020). Very mild isolated intellectual disability caused by adenylosuccinate lyase deficiency: A new phenotype. Mol. Genet. Metab. Rep..

[87] Kodera H., Ohba C., Kato M., Maeda T., Araki K., Tajima D., Matsuo M., Hino-Fukuyo N., Kohashi K., Ishiyama A. (2016). De novoGABRA1mutations in Ohtahara and West syndromes. Epilepsia.

[88] Sands T.T., Miceli F., Lesca G., Beck A.E., Sadleir L.G., Arrington D.K., Schönewolf-Greulich B., Moutton S., Lauritano A., Nappi P. (2019). Autism and developmental disability caused by KCNQ3 gain-of-function variants. Ann. Neurol..

[89] Smith L., Singhal N., El Achkar C.M., Truglio G., Sheidley B.R., Sullivan J., Poduri A. (2018). PCDH19 -related epilepsy is associated with a broad neurodevelopmental spectrum. Epilepsia.

[90] Kobayashi Y., Tohyama J., Kato M., Akasaka N., Magara S., Kawashima H., Ohashi T., Shiraishi H., Nakashima M., Saitsu H. (2016). High prevalence of genetic alterations in early-onset epileptic encephalopathies associated with infantile movement disorders. Brain Dev..

[91] Assoum M., Philippe C., Isidor B., Perrin L., Makrythanasis P., Sondheimer N., Paris C., Douglas J., Lesca G., Antonarakis S. (2016). Autosomal-Recessive Mutations in AP3B2, Adaptor-Related Protein Complex 3 β 2 Subunit, Cause an Early-Onset Epileptic Encephalopathy with Optic Atrophy. Am. J. Hum. Genet..

[92] Gorman K.M., Meyer E., Grozeva D., Spinelli E., McTague A., Sanchis-Juan A., Carss K.J., Bryant E., Reich A., Schneider A.L. (2019). Bi-allelic Loss-of-Function CACNA1B Mutations in Progressive Epilepsy-Dyskinesia. Am. J. Hum. Genet..

[93] Danti F.R., Galosi S., Romani M., Montomoli M., Carss K.J., Raymond F.L., Parrini E., Bianchini C., McShane T., Dale R.C. (2017). GNAO1 encephalopathy: Broadening the phenotype and evaluating treatment and outcome. Neurol. Genet..

[94] Venkatesan C., Angle B., Millichap J. (2016). Early-life epileptic encephalopathy secondary to SZT2 pathogenic recessive variants. Epileptic Disord..

[95] Vidal S., Brandi N., Pacheco P., Gerotina E., Blasco L., Trotta J.R., Derdak S., Del Mar O’Callaghan M., Garcia-Cazorla À., Pineda M. (2017). The utility of Next Generation Sequencing for molecular diagnostics in Rett syndrome. Sci. Rep..

[96] Sandweiss A.J., Brandt V.L., Zoghbi H.Y. (2020). Advances in understanding of Rett syndrome and MECP2 duplication syndrome: Prospects for future therapies. Lancet Neurol..

[97] Gillentine M.A., Wang T., Hoekzema K., Rosenfeld J., Liu P., Guo H., Kim C.N., De Vries B.B.A., Vissers L.E.L.M., Nordenskjold M. (2021). Rare deleterious mutations of HNRNP genes result in shared neurodevelopmental disorders. Genome Med..

[98] Ehrhart F., Coort S.L., Eijssen L., Cirillo E., Smeets E.E., Sangani N.B., Evelo C.T., Curfs L.M.G. (2020). Integrated analysis of human transcriptome data for Rett syndrome finds a network of involved genes. World J. Biol. Psychiatry.

